# Preliminary Independent Evaluation of Free2B: A Targeted Intervention to Promote the Mental Wellbeing of LGBTQIA+ Youth

**DOI:** 10.1002/jad.70035

**Published:** 2025-08-13

**Authors:** Qiqi Cheng, Neil Humphrey

**Affiliations:** ^1^ Manchester Institute of Education University of Manchester Manchester UK

**Keywords:** adolescent, intervention, LGBTQIA+, mentoring, propensity score matching

## Abstract

**Introduction:**

Young people who identify as LGBTQIA+ are significantly more likely to experience mental health problems than their cisgender, heterosexual peers. However, there is a dearth of evidence regarding interventions to support their wellbeing. This Short Research Article provides preliminary independent evidence of the efficacy of Free2B, a novel 1:1 mentoring service.

**Methods:**

A quasi‐experimental evaluation using propensity score matching and difference‐in‐differences analysis was used in a pre‐test/post‐test control group design to assess the impact of Free2B on the mental wellbeing (assessed via the Short Warwick‐Edinburgh Mental Wellbeing Scale, SWEMWBS). The intervention group consisted of *N* = 55 young people (aged 10–18, M = 14.5, SD = 2.0, 29.1% girl, 32.7% boy, 18.2% nonbinary, 3.6% describe in another way and 16.4% not sure), with matched control samples derived from a subset of the #BeeWell cohort study (*N* = 14, 715, aged 12–15, M = 13.2, SD = 0.7, 43.6% girl, 45.9% boy, 2.2% nonbinary, 3% describe in another way and 5.4% not sure). Data were collected in England between 2016 and 2023.

**Results:**

Participation in Free2B led to a statistically significant improvement in mental wellbeing (β = 3.81, 95% CI = [2.01, 5.61], d = 0.80). This effect was found to be robust across a series of sensitivity analyses (e.g., different matching methods, permutation tests).

**Conclusions:**

Free2B yields considerable promise as a means through which to improve the mental wellbeing of LGBTQIA+ young people. Accordingly, an explanatory trial is warranted.

## 1 Introduction

Research has consistently found that young people who identify as LGBTQIA+ are significantly more likely to experience mental health problems (e.g., anxiety, depression) than their cisgender, heterosexual peers (Connolly et al. 2016; Lucassen et al. 2017). Minority stress theory offers a compelling explanation, proposing that experiences of prejudice and discrimination, identity concealment, expectations of rejection and internalized stigma result in excess stress, which in turn leads to reduced wellbeing (Black et al. 2023).

Despite this, the volume and quality of research on the efficacy of targeted interventions is limited (McDermott et al. 2021). Indeed, a recent systematic review of psychotherapeutic interventions for LGBTQIA+ youth found only 10 studies, reporting that significant design and methodological limitations were present across most, with only two using any form of control group (Bochicchio et al. 2022). Similarly, a systematic review of psychosocial interventions for mental illness among LGBTQIA+ young people reported on nine studies, of which all but two were rated as having high risk of bias on at least one domain of the Cochrane Collaboration Risk of Bias tool (Van Der Pol‐Harney and McAloon 2019). Finally, a systematic review of empirically‐based psychological interventions for sexual minority youth found only eight studies, with again only two using any form of control group (Hobaica et al. 2018). Thus, while a number of interventions are reported to be efficacious, these claims are premature in the absence of a clear counterfactual and other methodological strengths (Van Der Pol‐Harney and McAloon 2019). Accordingly, there is a clear need for high quality intervention research that can help us to understand what works for this demonstrably vulnerable subset of the adolescent population.

The secondary data analysis reported herein provides preliminary evidence of the efficacy of Free2B, a 1:1 mentoring service based on the principles of information, advice and guidance. The name of the service was initially inspired by the 2014 London Pride theme (the year before the organization was founded) which was #FreedomTo, but was also informed by the notion that the service offers young people a safe space where they are free to be themselves.

Free2B has three core aims: improve wellbeing; build personal resilience; and, reduce isolation. Support is available to LGBTQIA+ young people aged 13–19+ (NB: though typically a 13–19 service, those aged 20–25 with special educational needs and disabilities can be referred if they meet very specific and individually assessed criteria, hence the “19 + ”). Referrals can be made directly by the young person, or via family, school or allied professionals. Free2B provides a safe space for young people to explore their situations and develop the necessary skills and strategies to manage the difficulties they face. Sessions are delivered by Community Support Workers (CSWs) in partner schools and community settings such as libraries or community centers, or online. CSWs are employed by Free2B, with essential recruitment criteria including experience of providing 1:1 targeted support to a caseload of young people, including undertaking assessments, planning, target setting and review (preferably in education settings); and, knowledge and experience of the key issues impacting on LGBTQIA+ young people. Desirable recruitment criteria include a qualification in a relevant area such as social work, community work, gender studies, teaching, and/or psychology.

Each young person's program is tailored to meet their specific needs. Frequency is typically weekly or fortnightly and usually 30–60 min long, but this is flexible. Session frequency may decrease as part of working towards a planned closure. Program length is open‐ended to ensure it meets each young person's individual needs. Goals are reviewed and updated every six to 10 sessions to ensure support remains meaningful and to avoid enabling overdependence. Examples of specific program foci include supporting a young person to: explore their sexuality/gender identity; understand practical transition steps (e.g., change of name process); cope with family rejection, or LGBTQIA + ‐related bullying; and/or seek LGBTQIA+ inclusive opportunities (e.g., further/higher education, employment).

We examine the impact of Free2B on mental wellbeing as it is a key theorized outcome of participation in the intervention. However, our focus was also driven by pragmatism (i.e., availability of a mental wellbeing measure common to both the Free2B and the #BeeWell datasets, the latter of which was used to derive a matched control group) and a recent call to expand the LGBTQIA+ youth intervention evidence base beyond mental health difficulties (Fish 2020). We hypothesized that *exposure to Free2B would lead to significant improvements in mental wellbeing of LGBTQIA+ youth, when compared to a propensity score matched control group of unexposed participants*.

## 2 Methods

### 2.1 Design and Participants

A pre‐test‐post‐test, difference‐in‐differences (DiD) design was employed to estimate the effect of Free2B on mental wellbeing. The study utilized data from two sources, the Free2B dataset and the #BeeWell dataset, comprising a total of *N* = 14,770 participants. The Free2B dataset, which contains information on individuals exposed to the Free2B intervention between 2016 and 2023, included 55 young people (M = 14.5, SD = 2.0, range = 10–18), whose mental wellbeing was assessed at baseline (T0) and at 9–15‐month post‐intervention follow‐up (T1). The #BeeWell dataset comprised *N* = 14,715 participants (M = 13.2, SD = 0.7, range = 12–15) from that study's longitudinal cohort who completed at least two waves of surveys between 2021 and 2023 (#BeeWell Research Team 2021). Ethical approval for the study was obtained from the University Research Ethics Committee (UREC) at the University of Manchester (ref: 2024‐16863‐37129).

### 2.2 Measures

#### 2.2.1 Mental Wellbeing

Mental wellbeing was measured using the Short Warwick Edinburgh Mental Wellbeing Scale (SWEMWBS) (Stewart‐Brown et al. 2009). This validated 7‐item self‐report instrument assesses both subjective and eudaimonic wellbeing (example item: “I've been feeling useful”). Participants rate each item on a five‐point scale (None of the time; Rarely; Some of the time; Often; All of the time). SWEMWBS was recently identified as an optimal wellbeing measure in a comprehensive analysis spanning assessment of dimensionality, measurement invariance, and convergent validity (Black et al. 2023). Internal consistency in the current study was excellent at both time points (T0 α = 0.87, T1 α = 0.89). Summed scores were used for analysis. This outcome was measured in both the Free2B and #BeeWell participants.

#### 2.2.2 Covariates

Participant socio‐demographic information included gender (girl [including trans girl], boy [including trans boy], nonbinary, I describe myself in another way, not sure), sexuality (pan/poly/omni sexual, bisexual; lesbian, gay; heterosexual; other, prefer not to say, asexual; questioning), special educational needs, ethnicity, and age.

#### 2.2.3 Additional Measures

Three additional measures available in the #BeeWell dataset were used to mimic Free2B treatment selection criteria: internalizing symptoms (scoring in the borderline or clinically significant range on the emotional difficulties subscale of the Me and My Feelings measure; Deighton et al. 2013); loneliness (reporting often or always feeling lonely on the Office for National Statistics' loneliness item; Office for National Statistics 2018); and, coping (scoring in the bottom 25% of the distribution of scores for the coping items in the Perceived‐Stress‐Scale‐4; Cohen et al. 1983).

### 2.3 Statistical Analyses

First, we used propensity score matching (PSM) to create a matched control group from the #BeeWell dataset that is comparable to the Free2B participants (the treated group) on observed pre‐intervention characteristics. Second, we used difference‐in‐differences (DiD) design on the matched sample to estimate the average treatment effect on the treated (ATT).

#### 2.3.1 Propensity Score Matching

To address potential selection bias arising from nonrandom assignment to Free2B, we employed PSM. This method aims to balance the distribution of observed covariates between the treated and control groups, thereby reducing the risk that observed differences in outcomes are due to pre‐existing differences rather than the treatment itself. Propensity scores, representing the estimated probability of participating in Free2B given observed pretreatment characteristics, were estimated using a logistic regression model. The model included the following pretreatment covariates: mental wellbeing; internalizing symptoms; sexuality; gender; loneliness; and, coping. The pretreatment covariates were selected based on two main principles. Firstly, based on theoretical considerations that these variables represent factors likely to influence a young person's selection into, or need for, the Free2B program, aligning with the program's selection criteria. Secondly, this selection was informed by information provided directly by Free2B regarding known characteristics or circumstances that typically lead young people to be referred to or seek out their service, thus influencing participation.

Age and ethnicity were not included in the propensity score model as they were not considered primary determinants of selection into Free2B and their inclusion led to a substantial reduction in the matched sample size without improving covariate balance. Finally, a specification test was conducted to assess the adequacy of this propensity score model (Sant'Anna and Song 2019). The null hypothesis, indicating that the model is correctly specified according to the test's criteria, was not rejected (*p* > 0.05 for both test statistics). This finding suggests that, based on this diagnostic check, our logistic model provides a reasonable approximation for the probability of participation given the included covariates, lending confidence to the PSM procedure's ability to balance these observed characteristics effectively between the treated and control groups.

Matching was performed using the *MatchIt* package (version 4.6.0) in R (version 4.3.3) (Ho et al. 2011). We used genetic matching with a caliper of 0.2 standard deviations of the logit of the propensity score. This caliper restricts potential matches to control units whose propensity scores are within 0.2 standard deviations of the treated unit's propensity score, ensuring reasonable common support and preventing poor matches. The target estimand was explicitly set to the average treatment effect on the treated (ATT). We also provide sensitivity analysis by presenting other matching methods, and results can be found in supplementary.

#### 2.3.2 Estimating the Treatment Effects

Following the creation of the matched sample, we employed a difference‐in‐differences (DiD) design to estimate the ATT. This approach compares the change in SWEMWBS scores from pre‐treatment to post‐treatment for the treated group (Free2B participants) to the corresponding change for the matched control group (untreated #BeeWell participants). The DiD estimator is obtained from the following two‐way fixed effects (TWFE) regression model:

$$y_{i,t}=\alpha_{i}+\phi_{t}+\beta_{\mathrm{treat}}D_{i,t}+1(t=\mathrm{post})X_{i,t=\mathrm{pre}}\beta_{\text{cov}}+\epsilon_{i,t}$$

where, the dependent variable, $y_{i,t}$ is the wellbeing outcome (SWEMWBS score) for individual i measured at time *t*. Our model includes individual fixed effect $\alpha_{i}$, time fixed effect $\phi_{t}$ and the indicator of treatment status of individual i previous to time *t*, $D_{i,t}$. For brevity, we have defined $D_{i,t}={\mathrm{Free}2B}_{i}\times 1\left(t=\mathrm{post}\right)$, where ${\mathrm{Free}2B}_{i}$ is a binary variable indicating whether individual i is in the Free2B program, and 1(t=post), which represents the post‐treatment dummy. The estimated $\hat{\beta_{\mathrm{treat}}}$ is the coefficient of interest, corresponding with the average treatment effect on the treated (ATT) under the parallel trends and no anticipation assumptions. $X_{i,t=\mathrm{pre}}$ is a vector of pre‐treatment control variables. This vector includes matched and any unmatched variables, for example, age, and ethnicity, to further adjust any remaining imbalance. Standard errors are clustered at the individual level for the full sample analysis and at the matched subclass level for the matched sample analysis.

The key identifying assumption for our DiD design is conditional parallel trends (CPT). In this context, we assume that, conditional on the matched covariates and the additional unmatched covariate, the average change in SWEMWBS scores in the absence of Free2B participation would have been the same for both the treated and matched control groups. We also impose a no‐anticipation assumption, requiring that measured outcomes in the pre‐treatment period (T0) were unaffected by future participation in the Free2B program.

## 3 Results

**Figure 1:**
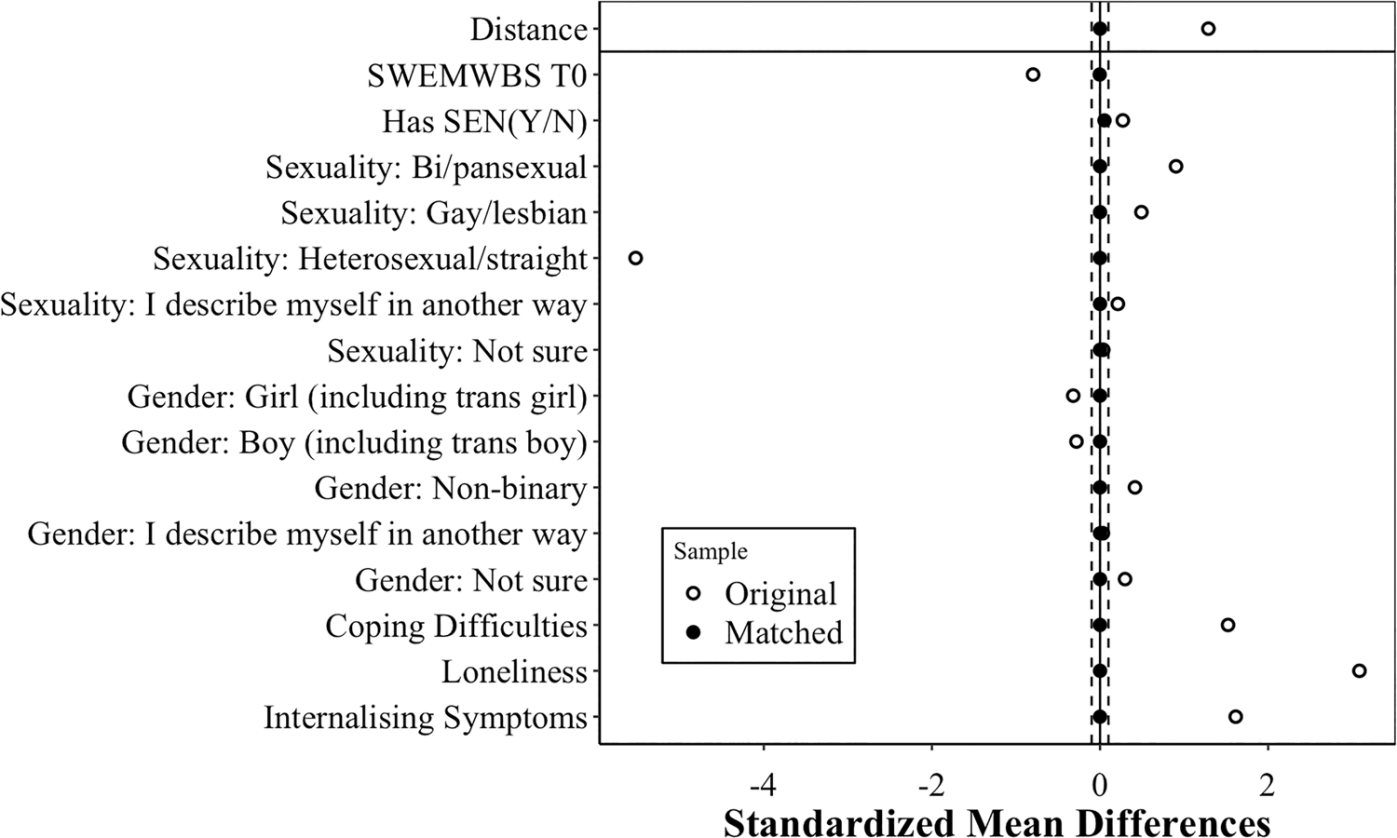
Covariate balance before and after propensity score matching. *Note:* The dashed lines show a 0.1 threshold for standardized mean differences.

Table S1 presents descriptive statistics for the Free2B (treated) and #BeeWell (potential control) samples before matching (see also Figure 1). As shown, there were significant differences between the groups on several pre‐treatment characteristics. For example, Free2B participants had lower average T0 SWEMWBS scores (M = 19.53, SD = 5.12) than the #BeeWell sample (M = 23.60, SD = 5.81). Significant differences were also observed for sexuality, coping difficulties, loneliness and internalizing symptoms. The standardized differences, shown in the 6th column, highlight the magnitude of these pre‐treatment imbalances. These initial imbalances suggest potential selection bias, motivating our use of PSM.

**Table 1:** Estimated effect of Free2B on SWEMWBS.

	Full sample			Prop. score matched sample		
	(1) TWFE	(2) TWFE	(3) Doubly Robust	(4) TWFE	(5) TWFE	(6) Doubly Robust
ATT(Free2B)	3.59*** (0.747)	3.76*** (0.637)	5.35*** (0.848)	3.55** (0.989)	3.81*** (0.950)	5.28*** (0.824)
Observations	29,540	29,540	29,540	180	180	180
R2	0.742	0.812	N/A	0.673	0.819	N/A
Within R2	0.001	0.275	N/A	0.096	0.498	N/A
Participants	14,715	14,715	14,715	90	90	90
Free2B participants	55	55	55	45	45	45
S.E. clustered	Individual	Individual	Analytical	subclass	subclass	Analytical
Core controls						
Individual fixed effect $\alpha_{i}$	Yes	Yes	N/A	Yes	Yes	N/A
Time fixed effect $\phi_{t}$	Yes	Yes	N/A	Yes	Yes	N/A
$1\left(t=\mathrm{post}\right)X_{i,t=\mathrm{pre}}$	No	Yes	N/A	No	Yes	N/A

*Note:* This table reports estimates using two approaches: a conventional Two‐Way Fixed Effects (TWFE) regression and the Doubly Robust Difference‐in‐Differences (DRDID) estimator following Sant'Anna and Zhao (2020). The outcome variable is the SWEMWBS raw score. Results in Columns 1–3 are based on the full sample comprising 55 Free2B participants and 14,715 control observations. Columns 4–6 utilize a matched sample (45 Free2B participants and 45 matched controls). For TWFE estimates, standard errors are clustered at the individual level for full sample and at the matched subclass level for the matched sample. The DRDID estimates use analytical standard errors derived from the influence function, consistent with Sant'Anna and Zhao (2020).

The matching process generated 45 matched pairs (*N* = 90) across the intervention and control groups (i.e., exclusion of 10 of 55 Free2B participants and 14,670 of 14,715 #Beewell participants). The standardized differences after matching were significantly reduced to below 0.1 for all covariates included in the propensity score model, indicating a substantial improvement in covariate balance (Franklin et al. 2014) (see Figure 1, Figure S1 and Table S1). Thus, PSM enabled the creation of a well‐matched control group for the covariates included in the propensity score model, suitable for subsequent DiD estimation. However, as expected, the variables not included in the matching process—age at pretest and ethnicity—remained imbalanced between the groups (Table S1). Given this remaining imbalance and to control for potentially differing trends based on all baseline characteristics, we subsequently employed a TWFE estimator including interaction terms between the post‐intervention period and the full set of baseline covariates.

We observed a statistically significant and positive impact of the Free2B intervention on SWEMWBS scores (β = 3.81, 95% CI [2.01, 5.61], Cohen's d = 0.80). This estimated effect proved robust across several specification checks (Table 1): it remained similar when excluding interaction terms between the post‐intervention period and baseline covariates in the TWFE, and when using the Doubly Robust Difference‐in‐Differences (DRDID) estimator (Sant'Anna and Zhao 2020). Furthermore, the positive effect persisted when applying the DRDID estimator to the full, unmatched sample (Table 1). Sensitivity analyses using alternative matching methodologies yielded comparable results (Table S2). Furthermore, estimates remained similar when including age at T0 and ethnicity in the propensity score model and performing matching without a caliper (see Table S3). Finally, a sensitivity analysis confirmed that our primary model's grouping of the gender variable was necessary, as an alternative model using a disaggregated gender variable (e.g., distinguishing between cisgender and transgender boys and girls) failed to achieve adequate covariate balance (see Table S4), a critical requirement for valid inference with propensity score matching (Stuart 2010).

To further assess the robustness of our findings and address potential concerns regarding unobserved heterogeneity, we employed a permutation‐based sensitivity analysis, randomly permuting Free2B intervention status (Free2B = 1) across the sample and re‐estimating the DiD model 10,000 times (La Ferrara et al. 2012). Since the “fake” Free2B = 1 variable is generated randomly, it should theoretically produce a nonsignificant estimate close to zero. If the DiD estimate was statistically significant, it would indicate that it had been mis‐specified. Using a randomly constructed Free2B = 1 variable, the estimates were centered around zero with a standard deviation of 1.14, suggesting no effect. Our benchmark estimate of 3.81, in contrast, was outside the 95% empirical interval (−2.24, 2.19), with a corresponding empirical *p*‐value < 0.001 (see Figure S2). These results provide compelling evidence that the observed positive and statistically significant impact of the Free2B intervention on the mental wellbeing of LGBTQIA+ youth is unlikely to be an artifact of unobserved confounding.

## 4 Discussion

Using DiD estimation and PSM as a robust, pragmatic alternative to random allocation through which causal inference is still possible, the current study demonstrated that participation in Free2B led to statistically significant improvement in mental wellbeing among LGBTQIA+ adolescents. This effect was found consistently across a series of sensitivity analyses (e.g., different matching methods, permutation tests). The observed effect size (3.81 points, equivalent to d = 0.80, or a 29‐percentile point increase) is meaningful and practically significant, being well above the threshold for statistically important change on SWEMWBS established in a responsiveness evaluation with a clinical sample (Shah et al. 2018). It also compares very favorably to the effect sizes observed in comparable LGBTQIA+ youth wellbeing intervention studies (e.g., d = 0.32–0.34 for mental health outcomes in Schwinn et al. 2015). Free2B therefore yields considerable promise as an efficacious intervention. Accordingly, an explanatory trial is warranted, to determine if the effect observed here can be replicated; assess the impact of the intervention on other theorized outcomes (e.g., isolation); examine treatment effect moderators (e.g., intervention compliance, contextual and environmental factors); and, ascertain if any observed effects are maintained in the longer‐term. Such research would enhance our understanding of the mechanisms underpinning Free2B's impact and inform evidence‐based improvements to the program design.

The current study had numerous strengths, including the use of DiD, PSM, and robustness and sensitivity checks. However, there are also limitations that need to be considered. First, there was some wastage ensuing from the PSM process (i.e., N = 10 of 55 excluded due to lack of adequate propensity score match in the #BeeWell dataset). Second, although the intervention and control groups were well matched on multiple pre‐treatment covariates (e.g., mental wellbeing; internalizing symptoms; sexuality; gender; loneliness; and, coping), other variables could influence longitudinal trends in mental wellbeing that were not included in the matching process (e.g., parent support). However, we were mindful of the need to avoid overfitting, and the matching process had to be pragmatic (i.e., contingent on data availability). Third, use of a design involving multiple pre‐ and post‐test measurements would have been preferable as this would have provided more robust evidence to support the parallel trends assumption in DiD than was possible in our simple pre‐test‐post‐test design. Finally, to align the pre‐ and post‐intervention measurement lag with the #BeeWell measurement cycle, the current study only considered young people who engaged with Free2B for 9–15 months. Our findings therefore do not necessarily apply to those who engage for longer or shorter periods.

Despite these limitations, the current study provides robust preliminary evidence that Free2B improves the mental wellbeing of LGBTQIA+ adolescents. Given the dearth of high‐quality intervention research, identifying what works for this demonstrably vulnerable subset of the adolescent population is an important step forward.

## Author Contributions

Qiqi Cheng and Neil Humphrey were responsible for the conceptualization and methodology of the study. Qiqi Cheng performed the formal analysis and validation of the data. Neil Humphrey was primarily responsible for writing – original draft preparation, and writing – review and editing, while Qiqi Cheng also contributed to writing – review and editing. Both authors had full access to the data, contributed to the interpretation of the results, and approved the final manuscript for submission. Qiqi Cheng takes responsibility for the integrity of the data and the accuracy of the data analysis.

## Ethics Statement

The study received ethical approval from the University Research Ethics Committee (UREC) at University of Manchester (ref number: 2024‐16863‐37129). Informed consent was obtained from the parents/legal guardians of all study participants included in the #BeeWell Project.

## Conflicts of Interest

The authors declare no conflicts of interest.

## Supporting information


**Appendix 1**: Balance of Covariates Before and After Matching.
**Appendix 2:** Sensitivity Analysis.

## Data Availability

The #BeeWell dataset used in the current study is available via protected access arrangements at the University of Manchester (UoM). The Free2B dataset is subject to a data sharing agreement (DSA) between UoM and Free2B and cannot be shared directly by the authors. Researchers interested in replicating the findings of the study will need to initiate a DSA with Free2B.
