# Supplementary material for: Preliminary Independent Evaluation of Free2B: A Targeted Intervention to Promote the Mental Wellbeing of LGBTQIA+ Youth
Source: J Adolesc. 2025 Aug 13;97(8):2273–8. doi: 10.1002/jad.70035 (PMC12682237; doi:10.1002/jad.70035)
Supplement: Supplementary file 1 — Appendix 1: Balance of Covariates Before and After Matching. Appendix 2: Sensitivity Analysis. [file JAD-97-2273-s001.docx]

**Preliminary independent evaluation of Free2B: a targeted intervention to promote the mental wellbeing of LGBTQIA+ youth: Supplementary Material**

**Appendix 1: Balance of Covariates Before and After Matching**

**Table S1.**

*Group Characteristics Before and After Propensity Score Matching*

|  |  | Original Unmatched Sample | | | |  | Matched Sample | | | |
| --- | --- | --- | --- | --- | --- | --- | --- | --- | --- | --- |
|  |  | Control | Treated | p | SMD |  | Control | Treated | p | SMD |
| N |  | 14715 | 55 |  |  |  | 45 | 45 |  |  |
| SWEMWBS T0 | Mean (SD) | 23.60 (5.81) | 19.53 (5.12) | <0.001 | 0.745 |  | 18.24 (4.05) | 18.22 (4.51) | 0.98 | 0.005 |
| Has SEN (%) | No | 12931 (87.9) | 42 (76.4) | 0.016 | 0.304 |  | 36 (80.0) | 35 (77.8) | 1 | 0.054 |
|  | Yes | 1784 (12.1) | 13 (23.6) |  |  |  | 9 (20.0) | 10 (22.2) |  |  |
| Sexuality(%) | Bi/pansexual | 1120 (7.6) | 29 (52.7) | <0.001 | 2.544 |  | 22 (48.9) | 22 (48.9) | 1 | <0.001 |
|  | Gay/lesbian | 401 (2.7) | 13 (23.6) |  |  |  | 10 (22.2) | 10 (22.2) |  |  |
|  | Heterosexual/straight | 11129 (75.6) | 1 (1.8) |  |  |  | 1 (2.2) | 1 (2.2) |  |  |
|  | Describe in another way | 634 (4.3) | 6 (10.9) |  |  |  | 6 (13.3) | 6 (13.3) |  |  |
|  | Not sure | 1431 (9.7) | 6 (10.9) |  |  |  | 6 (13.3) | 6 (13.3) |  |  |
| Gender (%) | Girl (including trans girl) | 6412 (43.6) | 16 (29.1) | <0.001 | 0.727 |  | 13 (28.9) | 13 (28.9) | 1 | <0.001 |
|  | Boy (including trans boy) | 6755 (45.9) | 18 (32.7) |  |  |  | 13 (28.9) | 13 (28.9) |  |  |
|  | Non-binary | 317 (2.2) | 10 (18.2) |  |  |  | 9 (20.0) | 9 (20.0) |  |  |
|  | Describe in another way | 442 (3.0) | 2 (3.6) |  |  |  | 1 (2.2) | 1 (2.2) |  |  |
|  | Not sure | 789 (5.4) | 9 (16.4) |  |  |  | 9 (20.0) | 9 (20.0) |  |  |
| Coping Difficulties (%) | No | 10296 (70.0) | 0 (0.0) | <0.001 | 2.159 |  | 0 (0.0) | 0 (0.0) | NA | <0.001 |
|  | Yes | 4419 (30.0) | 55 (100.0) |  |  |  | 45 (100.0) | 45 (100.0) |  |  |
| Loneliness (%) | No | 13357 (90.8) | 0 (0.0) | <0.001 | 4.435 |  | 0 (0.0) | 0 (0.0) | NA | <0.001 |
|  | Yes | 1358 (9.2) | 55 (100.0) |  |  |  | 45 (100.0) | 45 (100.0) |  |  |
| Internalising Symptoms (%) | No | 10651 (72.4) | 0 (0.0) | <0.001 | 2.289 |  | 0 (0.0) | 0 (0.0) | NA | <0.001 |
|  | Yes | 4064 (27.6) | 55 (100.0) |  |  |  | 45 (100.0) | 45 (100.0) |  |  |
| Ethnicity | Asian | 2430 (16.5) | 8 (14.5) | 0.187 | 0.38 |  | 2 (4.4) | 6 (13.3) | 0.108 | 0.668 |
|  | Black | 721 (4.9) | 3 (5.5) |  |  |  | 1 (2.2) | 1 (2.2) |  |  |
|  | Mixed | 847 (5.8) | 7 (12.7) |  |  |  | 2 (4.4) | 7 (15.6) |  |  |
|  | Other | 346 (2.4) | 3 (5.5) |  |  |  | 1 (2.2) | 3 (6.7) |  |  |
|  | White | 9977 (67.8) | 34 (61.8) |  |  |  | 37 (82.2) | 28 (62.2) |  |  |
|  | Chinese | 164 (1.1) | 0 (0.0) |  |  |  | 0 (0.0) | 0 (0.0) |  |  |
|  | Unclassified | 230 (1.6) | 0 (0.0) |  |  |  | 2 (4.4) | 0 (0.0) |  |  |
| Age T0 | Mean (SD) | 13.15 (0.72) | 14.53 (1.99) | <0.001 | 0.923 |  | 13.16 (0.77) | 14.47 (2.11) | <0.001 | 0.827 |

Note. For binary variables, p-values are obtained from Pearson's chi-squared test with continuity correction and SMD is the multivariate Mahalanobis distance between group-specific proportions. For continuous variables, p-values are obtained from one-way ANOVA assuming equal variance. The statistics are obtained using the R package *tableone*. SEN = Special education needs. SWEMWBS = Short Warwick-Edinburgh Mental Wellbeing Scale score. T0 = Pretreatment. N = number of observations. Ethnicity and Age are not included in the propensity score model as they are not considerate primary determinants of selection into Free2B. The identical distributions for gender and sexuality in the matched sample are a result of the large control pool, which allowed for exact matching on these categorical variables.


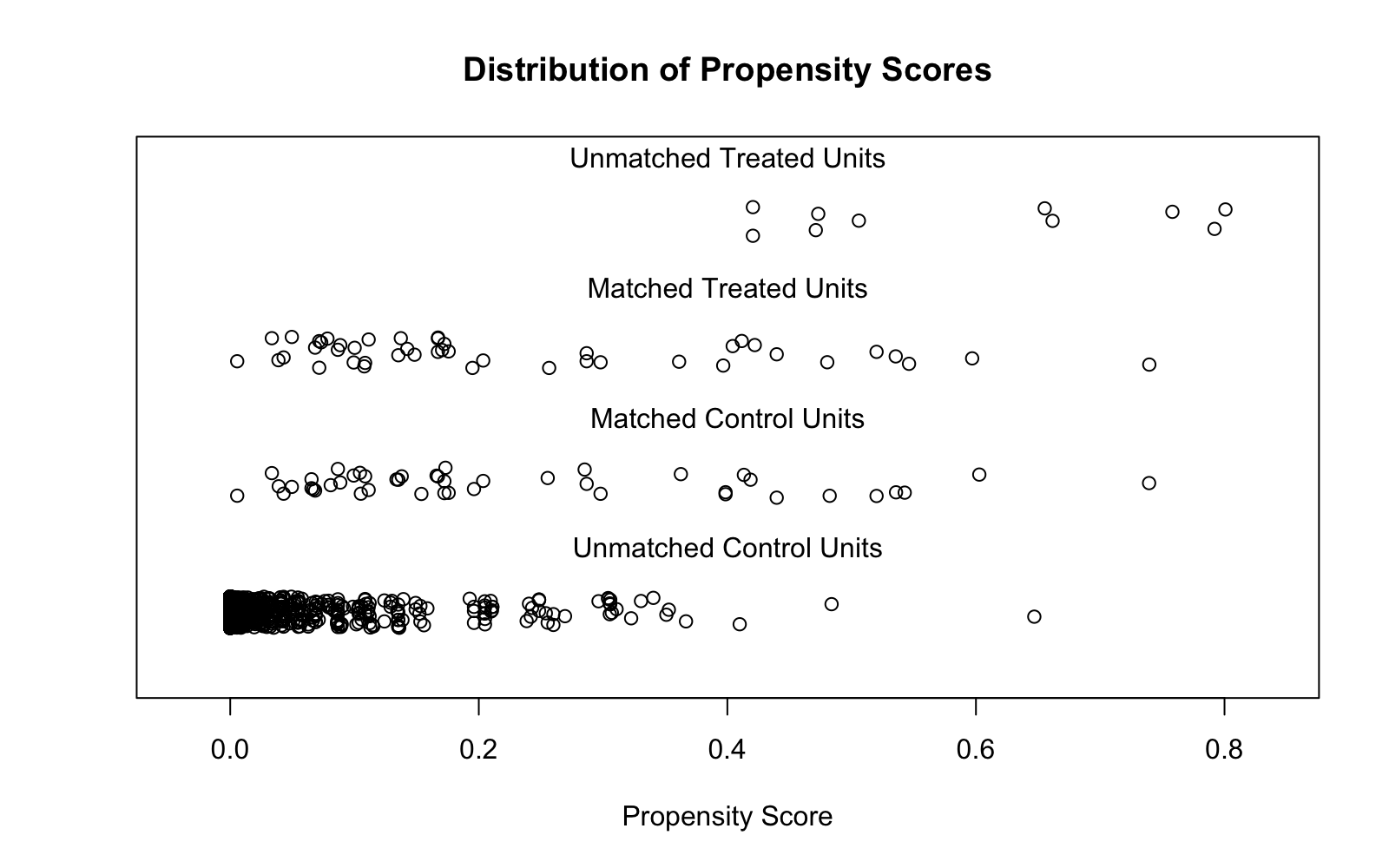


**Figure S1**.

*Jitter Plot Demonstrating the Distribution of Propensity Scores in Matched and Unmatched Samples*

**Appendix 2: Sensitivity Analysis**

**Table S2**
*Estimated intervention effects obtained from alternative matching methods.*

| Matching | Unbalanced | | N | | SWEMWBS T0 | | SWEMWBS T1 | | $\beta$ | *SE* | *t* | *p* | *d* |
| --- | --- | --- | --- | --- | --- | --- | --- | --- | --- | --- | --- | --- | --- |
|  | raw | std | Treated | Control | Treated | Control | Treated | Control |  |  |  |  |  |
| NN1 | 0 | 1 | 41 | 41 | 17.805 | 17.756 | 22.764 | 18.634 | 5.130 | 0.862 | 5.949 | 0.000 | 1.021 |
| NN5 | 0 | 2 | 41 | 115 | 17.805 | 16.983 | 22.764 | 17.957 | 5.350 | 0.851 | 6.290 | 0.000 | 1.013 |
| NN10 | 0 | 2 | 41 | 155 | 17.805 | 16.806 | 22.764 | 17.697 | 5.387 | 0.871 | 6.188 | 0.000 | 1.042 |
| Full | 0 | 0 | 40 | 14643 | 17.825 | 23.644 | 22.042 | 23.589 | 1.961 | 1.138 | 1.724 | 0.089 | 0.335 |
| Optimal | 2 | 7 | 55 | 55 | 19.527 | 18.509 | 23.079 | 18.782 | 4.177 | 0.890 | 4.692 | 0.000 | 0.802 |
| Exact | 0 | 0 | 21 | 44 | 17.762 | 17.545 | 23.238 | 19.159 | 5.163 | 1.716 | 3.009 | 0.005 | 1.114 |
| CEM | 0 | 0 | 27 | 78 | 18.111 | 18.064 | 23.562 | 19.526 | 4.873 | 1.213 | 4.018 | 0.000 | 1.050 |
| NN1R | 0 | 4 | 42 | 37 | 17.976 | 17.649 | 22.817 | 18.568 | 5.043 | 1.003 | 5.025 | 0.000 | 0.990 |

Note. Unbalanced = number of unbalanced covariates in the matched sample. N = number of observations in the matched sample. T0, T1 = Time 0, 1. *b* = the effect. NN5 = 1:5 Nearest neighbour matching. NN1R = 1:1 Nearest neighbour matching with replacement. CEM = Coarsened Exact Matching.

**Table S3**
*Estimated Intervention Effects from an Alternative Propensity Score Model Including All Observed Covariates and Matching Without a Caliper.*

| Matching | Unbalanced | | N | | SWEMWBS T0 | | SWEMWBS T1 | | $\beta$ | *SE* | *t* | *p* | *d* |
| --- | --- | --- | --- | --- | --- | --- | --- | --- | --- | --- | --- | --- | --- |
|  | raw | std | Treated | Control | Treated | Control | Treated | Control |  |  |  |  |  |
| NN1 | 3 | 8 | 55 | 55 | 19.527 | 19.145 | 23.079 | 18.364 | 4.684 | 0.939 | 4.988 | 0.000 | 0.894 |
| NN5 | 3 | 8 | 55 | 275 | 19.527 | 16.171 | 23.079 | 18.004 | 4.555 | 0.965 | 4.722 | 0.000 | 0.875 |
| NN10 | 3 | 8 | 55 | 550 | 19.527 | 15.755 | 23.079 | 18.218 | 4.492 | 1.038 | 4.329 | 0.000 | 0.847 |
| Genetic | 6 | 11 | 55 | 55 | 19.527 | 19.400 | 23.079 | 18.545 | 4.382 | 0.941 | 4.656 | 0.000 | 0.860 |
| Full | 7 | 14 | 55 | 14715 | 19.527 | 23.604 | 23.079 | 23.560 | 6.562 | 1.286 | 5.102 | 0.000 | 1.117 |
| Optimal | 5 | 11 | 55 | 55 | 19.527 | 19.000 | 23.079 | 18.636 | 4.417 | 0.917 | 4.819 | 0.000 | 0.840 |
| NN1R | 8 | 15 | 55 | 27 | 19.527 | 20.074 | 23.079 | 17.556 | 6.725 | 1.337 | 5.029 | 0.000 | 1.211 |

Note. Unbalanced = number of unbalanced covariates in the matched sample. N = number of observations in the matched sample. T0, T1 = Time 0, 1. T1_ Treated = Weighted SWEMWBS in the treated group at T1. T1_ Control = Weighted SWEMWBS in the matched control group at T1. *b* = the effect. NN5 = 1:5 Nearest neighbour matching. NN1R = 1:1 Nearest neighbour matching with replacement. CEM = Coarsened Exact Matching.

**Table S4**

*Estimated Intervention Effects from Sensitivity Analysis Using a Disaggregated Gender Variable*

|  |  | Full sample | | |  | Prop. Score matched sample | | |
| --- | --- | --- | --- | --- | --- | --- | --- | --- |
|  |  | (1) TWFE | (2) TWFE | (3) Doubly Robust |  | (4) TWFE | (5) TWFE | (6) Doubly Robust |
| ATT(Free2B) |  | 3.60*** (0.747) | 3.98***  (0.651) | 4.56*** (0.756) |  | 5.10*** (0.950) | 5.30*** (0.984) | 4.20*** (1.151) |
|  |  |  |  |  |  |  |  |  |
| Observations |  | 29,530 | 29,530 | 29,530 |  | 148 | 148 | 148 |
| R2 |  | 0.742 | 0.813 | N/A |  | 0.76086 | 0.84925 | N/A |
| Within R2 |  | 0.001 | 0.275 | N/A |  | 0.21066 | 0.50240 | N/A |
| Participants |  | 14,710 | 14,710 | 14,710 |  | 74 | 74 | 74 |
| Free2B Participants |  | 55 | 55 | 55 |  | 37 | 37 | 37 |
| S.E. Clustered |  | Individual | Individual | Analytical |  | subclass | subclass | Analytical |
|  |  |  |  |  |  |  |  |  |
| Core controls |  |  |  |  |  |  |  |  |
| Individual fixed effect $\alpha_{i}$ |  | Yes | Yes | N/A |  | Yes | Yes | N/A |
| Time fixed effect $\phi_{t}$ |  | Yes | Yes | N/A |  | Yes | Yes | N/A |
| $1\left( t=post \right)\boldsymbol{X}_{i, t=pre}$ |  | No | Yes | N/A |  | No | Yes | N/A |

Notes: This table reports estimates from a sensitivity analysis where the gender variable was disaggregated to distinguish between cisgender and transgender participants. Estimates were generated using two approaches: a conventional Two-Way Fixed Effects (TWFE) regression and the Doubly Robust Difference-in-Differences (DRDID) estimator following Sant'Anna and Zhao (2020). The outcome variable is the SWEMWBS raw score.

Results in Columns 1-3 are based on the full sample (55 Free2B participants; 14,715 control observations). Columns 4-6 utilize a propensity score matched sample (37 Free2B participants; 37 matched controls).

Critically, the propensity score matching process using this disaggregated gender variable failed to achieve adequate covariate balance. Several standardized mean differences (SMDs) exceeded the conventional 0.1 threshold, indicating significant remaining imbalance between the groups. Key imbalances were observed for: Gender (Not sure, SMD = 0.173); Sexuality (Trans girl, SMD = 0.238); Sexuality (Other/Prefer not to say, SMD = -0.144).

The poor balance in this sensitivity analysis supports the decision to use the original, grouped gender variable in the primary analysis presented in the main manuscript to ensure methodological rigor.

For TWFE estimates, standard errors are clustered at the individual level for the full sample and at the matched subclass level for the matched sample. The DRDID estimates use analytical standard errors derived from the influence function, consistent with Sant'Anna and Zhao (2020).

**Appendix 3: Robustness Check**


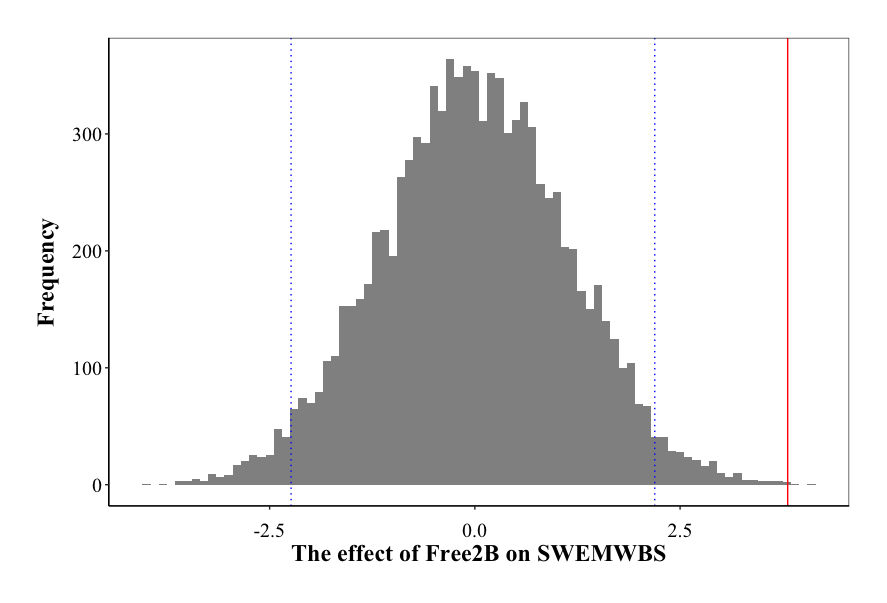


*Figure S2. Distribution of Coefficients in the Falsification Permutation Test / Note. The graph illustrates the empirical density of estimated coefficients obtained from 10000 simulations wherein the Free2B status was randomly assigned to participants within the matched data set. The red solid vertical line represents the effect reported in Column 5 in Table 1. The dashed blue line represents the 95% CI of the empirical distribution in this falsification test.*
